# Developing a Novel Scoring System for Risk Stratification in Living Donor Liver Transplantation

**DOI:** 10.3390/jcm10092014

**Published:** 2021-05-08

**Authors:** Hao-Chien Hung, Chen-Fang Lee, Ssu-Min Cheng, Wei-Chen Lee

**Affiliations:** 1 Department of Liver and Transplantation Surgery, Chang-Gung Memorial Hospital at Linkou, Taoyuan City 333, Taiwan; mp0616@cgmh.org.tw (H.-C.H.); weichen@cgmh.org.tw (W.-C.L.); 2 College of Medicine, Chang-Gung University, Taoyuan City 333, Taiwan; 3 Nursing Department, Chang-Gung Memorial Hospital at Linkou, Taoyuan City 333, Taiwan; qsimin@gmail.com

**Keywords:** risk stratification, scoring system, living donor liver transplantation

## Abstract

Background: We aimed to develop a novel scoring system for risk stratification specific to living donor liver transplantation (LDLT) recipients, to improve the accuracy of predicting short-term outcomes. Methods: The sequential organ failure assessment (SOFA) score at postoperative day 7 was collected and simplified by dichotomization, and these categories and other clinical factors were subjected to univariate and multivariate logistic regression analyses to select independent risks in constructing a “graft-to-recipient weight ratio (GRWR)-SOFA” scoring system. Results: We enrolled 519 patients who underwent LDLT. The GRWR-SOFA score comprises a sum of six factors: cardiovascular (mean arterial pressure < 70 mmHg, scored 3), coagulation (serum platelet < 50 × 10^3^/μL, scored 2), renal (creatinine > 1.2 mg/dL, scored 2), liver (serum total bilirubin > 5.9 mg/dL, scored 5), neurological (Glasgow coma scale < 15, scored 2), and GRWR < 0.8, scored 2. The GRWR-SOFA contained four classes: The early mortality rate at 3 months and 1 year after LDLT was 1.3% and 6.9% for class I (scores of 0–4), 9.1% and 16.7% for class II (scores of 5–8), 25.5% and 34% for class III (scores of 9–10), and 61.3% and 67.7% for class IV (scores ≥ 11), respectively. The area under the receiver operating characteristic curve of GRWR-SOFA in the 3-month mortality prediction was 0.881 (95% confidence interval (CI): 0.818–0.944). Conclusions: The GRWR-SOFA model demonstrates superior discriminatory power for predicting short-term mortality. It enables clinicians to identify the right level of care for distinct subgroups of patients receiving LDLT.

## 1. Introduction

Liver transplantation (LT) is the treatment of choice for patients with acute liver failure, end-stage liver disease, or hepatocellular carcinoma. Owing to the lack of deceased donors, the number of living donor liver transplantation (LDLT) cases are increasing, with significant improvement in surgical techniques and surgeon-experiences [1,2]. Although LDLT has markedly advanced during the past decades, some recipients might still be confronted with adverse outcomes, especially in the early post-transplant period. Therefore, clinicians have developed scoring systems or models that could predict adverse outcomes or mortality early after LT [3,4]. Compared with deceased donor LT, the associated factors in determining post-LDLT outcomes are more multisystemic and complex. For instance, the graft-to-recipient weight ratio (GRWR) is one of the major concerns in LDLT.

Being one of the most commonly used models for critically ill patients, the sequential organ failure assessment (SOFA) scoring system [5] consists of multisystem parameters, allowing for evaluation of organ deterioration and risk assessment. Our group has earlier reported that the SOFA score on post-operative day (POD) 7 with a cut-off value of 7 has successfully discriminated survivors and non-survivors at the early post-transplant period [6]. However, the SOFA score was developed from a general population rather than living donor recipients, and the relationship between each component and the liver allograft function has not been well assessed. Furthermore, a dichotomous method (≤7 or >7) could probably under- or overestimate the risk for patients with borderline scores.

In this study, we tried to validate the SOFA scores for a larger population and analyze each component of this scoring system for determining their weights in predicting survival. The ultimate aim of this study was to develop a novel scoring system for risk stratification that is specific for LDLT recipients to improve the accuracy of predicting short-term outcomes.

## 2. Materials and Methods

### 2.1. Study Population and Data Collection

This study was conducted at the Chang Gung Memorial Hospital in Taiwan. The hospital ethics committee approved this study (CGMH IRB No. 202001325B0). Consecutive liver transplant cases were retrospectively collected and reviewed during the period of January 2010 and March 2019. After excluding pediatric patients (*n* = 3), those who received deceased donor liver transplant (*n* = 164), and those lacking essential information that necessitated further analyses (*n* = 5), we finally enrolled 519 patients with LDLT. The techniques used in LDLT were performed using standard procedures as described in our previously published study [7]. Regarding post-transplant care, the protocols of respiration, coagulation, hemodynamic, and metabolic support in the intensive care unit were kept consistent. A checklist consisting of three aspects was extracted: general information (including recipient age, body mass index, sex, liver function, and etiology), transplant parameters (including donor and graft information, GRWR, surgical details), and SOFA categories at POD 7. The primary outcome assessed was 3-month mortality after transplant, while the secondary outcome was 1-year mortality.

### 2.2. SOFA Score Calculation and SOFA Component Dichotomy

The SOFA score [5] at POD 7 was calculated by adding corresponding scores from the extension of vital organ function and rate of failure. Traditionally, the allocated scores in the six vital functions of respiration, coagulation, liver, cardiovascular, renal, and neurologic systems were calculated on a scale of 0 to 4 based on the definition of the SOFA scoring system [5]. For unravelling and simplifying each SOFA component, we dichotomized each scale by the exact point in which the median case was located (Supplemental Table S1). We applied median split in the current study, because there may exist distinct groups of individuals and it makes subsequent analyses more straightforward and readily understood. Specific cut-off values were given in each SOFA category in the following order: 0 for cardiovascular component, 2 for coagulation component, 0 for renal component, 2 for liver component, 0 for neurologic component, and 1 for respiratory component. These modified parameters also gave robust statistical characteristics and avoided an arbitrarily low or high score distribution.

### 2.3. Statistical Analysis

Qualitative parameters (expressed as numbers and percentages) were analyzed by Pearson’s chi-square test, while quantitative parameters (mean ± standard deviations or median with ranges) were compared by independent *t*-test or non-parametric tests between post-transplant 3-month survivors and non-survivors. Potential factors in univariate logistic regression analysis with *p*-value < 0.100 were recognized and enlisted into the multivariate model to filter independent risk factors for post-transplant 3-month mortality. Calibration and discrimination performance of predictive models were examined using the Hosmer–Lemeshow method [8] and the area under curve (AUC) under the receiver operating characteristic (ROC) with 95% confidence interval (CI), respectively. The superiority over different ROC curves was determined using the DeLong test [9], while easyROC [10] was used for the statistical analysis. Statistical significance was defined as a two-tailed *p* value of <0.050. Survival differences depending on risk classification were assessed using the Kaplan–Meier method and log-rank test. Analyses and figures were performed using SPSS Statistics version 24.0 (SPSS Inc., Chicago, IL, USA).

## 3. Results

### 3.1. Characteristics of Enrolled Patients

Table 1 shows the general information of the recipients and donors, transplant-associated factors, and original SOFA score with corresponding components on POD 7. A total of 519 recipients with a mean age of 53.9 ± 8.8 (19.1–70.2) years, majority being males (74.8%), who underwent LDLT were enrolled. The leading etiology was viral hepatitis (73.6%), while 43.7% had hepatocellular carcinoma. There were 119 (22.9%) patients with a history of alcohol use. There were 198 (38.2%) cases classified as Child–Pugh class C, and the mean model for end-stage liver disease (MELD) score was 16.9 ± 8.9 (8–40). The average age of the donors was 32.3 ± 9.2 (18.1–59.3) years. Most of the allografts were right liver with a mean GRWR of 0.98 ± 0.25 (0.51–2.02). The mean surgical time of LDLT was 630.9 ± 140.0 min, ranging from 428.0–1219.0 min. The total SOFA scores at POD 7 of all included patients were 5.5 ± 2.8 (0–16).

### 3.2. Comparison between the Survivor and Non-Survivor Groups

Of the total of 519 patients who underwent LDLT, 42 (8.1%) patients died within 3 months after transplantation, referred to as the non-survivor group, and the remaining 477 patients were classified into the survivor group. The comparison of clinical variables and dichotomized SOFA components between the two groups is summarized in Table 2. Based on the comparison to patients in the survivor group, non-survivors were at more advanced cirrhotic classification (*p* = 0.008). Moreover, the MELD scores before transplantation were significantly higher in the non-survivor group (*p* = 0.013). The prevalence of viral hepatitis (*n* = 358, 51.7% vs. *n* = 24, 75.1%, *p* = 0.012) and liver cancer (*n* = 217, 23.8% vs. *n* = 10, 45.5%, *p* = 0.007) was lower in the non-survivor group compared to the survivor group, and a trend in alcohol use (*n* = 106, 22.2% vs. *n* = 13, 31.0%, *p* = 0.197) was observed in the non-survivor group. Although there was no difference in recipient age, donor age was different (32.0 ± 9.0 vs. 36.6 ± 9.8, *p* = 0.002) between the two groups. Several unfavorable perioperative factors, including less GRWR (*p* < 0.001), higher blood loss > 3000 mL (*p* = 0.012), ascites amount > 3000 mL (*p* = 0.002), and longer duration of cold ischemic time (*p* = 0.035), were correlated with early mortality within 3 months. The median SOFA score at POD 7 was 5 and 9 in the survivor and non-survivor groups, respectively (*p* < 0.001). A substantially higher proportion (*n* = 30, 71.4%) of the non-survivors were defined as high-risk according to the previous definition (SOFA > 7 at POD 7) [6]. Notably, near all numbers of re-dichotomized SOFA categories, the respiration component resembled that of a higher category indicated in early mortality after transplant.

### 3.3. Validation of SOFA Scores at POD 7

To validate the SOFA score at POD 7 for enrolled subjects, we applied Hosmer–Lemeshow’s goodness of fit test and obtained a *p*-value of 0.72, which indicated a comparable calibration result. The performance of the SOFA score at POD 7 to predict the 3-month mortality was illustrated with an AUC of 0.859 (95% CI: 0.799–0.920; Supplemental Figure S1A). An optimal cut-off value of 7 was used, and it demonstrated an AUC of 0.782 (95% CI: 0.700–0.854; Supplemental Figure S1B), and a significant increase in 3-month mortality was observed when the score was >7. With a cut-off value of 7, the SOFA model yielded a sensitivity of 71.4% and specificity of 84.9%. These data supported the effectiveness of SOFA at POD 7 in predicting post-transplant early mortality.

### 3.4. Independent Risks for Predicting Mortality within 3 Months after Transplant

The clinical parameters including dichotomized SOFA components were subjected to univariate analysis and those significant (*p* < 0.100) were entered into multivariate analysis with backward selection to identify independent risks for 3-month mortality. In univariate analysis, of all the available clinical factors considered, twelve factors were significantly associated with the 3-month mortality after transplant, as shown in Table 3. These potential factors were entered in the multivariate analysis that subsequently confirmed GRWR < 0.8 (hazard ratio (HR) = 3.11; 95% CI = 1.40–6.89; *p* = 0.005) and higher components of cardiovascular (HR = 5.31; 95% CI = 1.62–17.49; *p* = 0.006), coagulation (HR = 2.64; 95% CI = 1.12–6.21; *p* = 0.026), renal (HR = 2.69; 95% CI = 1.23–5.86; *p* = 0.013), liver (HR = 9.63; 95% CI = 3.92–23.66; *p* < 0.001), and neurologic (HR = 2.75; 95% CI = 1.15–6.61; *p* = 0.024) as independent risks for 3-month mortality.

### 3.5. Development of a Novel Scoring Model

**Figure 1:**
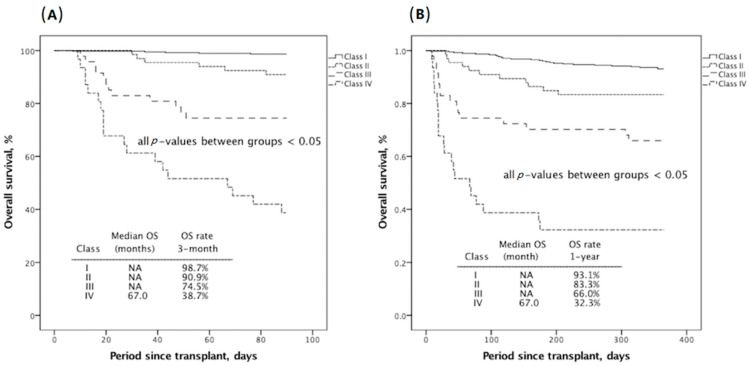
Kaplan–Meier survival comparison of 3-month (**A**) and 1-year (**B**) overall survival according to classified graft-to-recipient weight ratio-sequential organ failure assessment (GRWR-SOFA) risk scoring model. As the GRWR-SOFA class increases, the corresponding survival rate decreases.

Next, the factors that influenced 3-month mortality in multivariate logistic regression were given relative score allocation, which was transformed by multiplying the beta coefficients by two and then rounding to an integer. For computation of a new score, the weight of each independent risk was calculated and set by reference to “The Framingham Study risk score” as described by Sullivan et al. [11]. Consequently, a sum of six independent risks, namely the cardiovascular component representing mean arterial pressure < 70 mmHg (given score: 3), coagulation component for serum platelet < 50 × 10^3^/μL (given score: 2), renal component for creatinine > 1.2 mg/dL (given score: 2), liver component for serum total bilirubin > 5.9 mg/dL (given score: 5), neurologic component for Glasgow coma scale < 15 (given score: 2), and GRWR < 0.8 (given score: 2) (Table 4). The complete score of this novel system contains 16 points. By possible permutation, there would be no case with a sum score of 1 and 15. None of our patients fulfilled a score of 16 (Supplemental Table S2). Subsequently, we divided all into eight groups by every two scores in sequence (0, 2, 3–4, 5–6, 7–8, 9–10, 11–12, and 13–14) because of unit-digit case numbers in scores of 3, 6, 8, 10, 12, 13, and 14. The correlated 3-month mortality probability for each group is shown in Supplemental Table S2. We compared the Kaplan–Meier survival in each group to create a valid classification, and merged similar eventful probabilities using the following rules: (1) for groups in the same classification, the 3-month survival did not differ, and (2) the highest score group displayed a better trend in survival probability compared to the lowest-scoring group in the next classification (*p* < 0.100, Supplemental Figure S2). At the end of this process, class I (very low risk), class II (low risk), class III (intermediate risk), and class IV (high risk) corresponded to scores of 0–4, 5–8, 9–10, and ≥11, respectively (Table 4). There were 375 (72.3%) patients in the GRWR-SOFA class I, 66 (12.7%) in class II, 47 (9.1%) in class III, and 31 (6.0%) patients in class IV. As shown in Figure 1, the early mortality events at 3 months and 1 year after surgery were 1.3% and 6.9% for class I, 9.1% and 16.7% for class II, 25.5% and 34% for class III, and 61.3% and 67.7% for class IV, respectively (all *p* < 0.05 between any two classes). The newly developed “GRWR-SOFA” assessment tool also succeeded in predicting 1-year survival difference between each class (all *p* < 0.05 between any two classes, Figure 1B).

### 3.6. Performance Assessment of the Novel GRWR-SOFA Model

Distribution of the GRWR-SOFA model reclassified subjects compared to the original SOFA model is shown in Supplemental Table S3. In the original SOFA score ≤ 7 category, 417 patients and 24 (5.7%) patients were reclassified as GRWR-SOFA higher class III and IV, respectively. Among these 24 patients, five (20.8%) had early mortality. In contrast, with an original SOFA score >7, a total of 102 patients were considered to be at risk of early post-transplant mortality. Forty-eight patients were reclassified into class I or II, and 4 (8.3%) of them died within 3 months.

**Figure 2:**
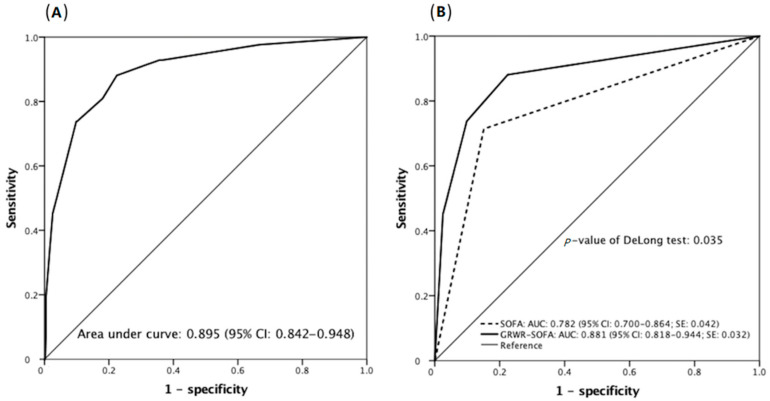
(**A**) Receiver operating characteristic for the graft-to-recipient weight ratio-sequential organ failure assessment (GRWR-SOFA) score affecting 3-month mortality after transplant. (**B**) The newly developed GRWR-SOFA model demonstrates a better discriminative power than the original SOFA model (*p*-value of the DeLong test = 0.035).

The ROC of our GRWR-SOFA system in the 3-month mortality prediction before stratification had an AUC of 0.895 (95% CI: 0.842–0.948; Figure 2A), and the AUC after stratification was 0.881 (95% CI: 0.818–0.944; Figure 2B). The correlated sensitivity/specificity was 88.1%/77.6%, 73.8%/90.2%, and 45.2%/97.5% for cut-off values of 4, 8, and 10, respectively. The discrimination ability between our GRWR-SOFA and original SOFA models was assessed using the DeLong test, which indicated that the GRWR-SOFA was a superior prediction model with respect to the original SOFA (*p* = 0.035, Figure 2B).

## 4. Discussion

A highly significant impact of post-transplant SOFA scores on prognosis after liver transplantation is shown. The present study supported that SOFA at POD 7 remains effective in predicting post-transplant early mortality after a decade. A full SOFA score is constituted by multisystemic measurement. We assumed that the SOFA components were not equal in predicting early mortality, and re-determined their weights in multivariate analysis. Our study’s greatest strength came from the modification of simplified SOFA components; it not only allows convenient score calculation but also emphasizes different vital organ weights in post-transplant intensive care and adverse outcome prediction exclusively. The functionality and discriminative ability of our novel GRWR-SOFA model has been tested, with demonstration of excellent results. This stratified model may assist in making logical management decisions and match risk with the level of care.

The GRWR-SOFA model consists of GRWR instead of the respiratory component. A GRWR < 0.8% should be considered an indicator of negative short-term outcomes. The use of a safe ratio range of GRWR > 0.8% has been generally acknowledged as an important predictor of the adequacy of post-transplant liver function. There appears to be a reduction in overall survival with a GRWR < 0.8% in LDLT, especially in patients with an elevated portal vein pressure >20 mmHg [12,13]. However, the rule is not absolute and exceptions in small GRWR with fair graft function achievement have been reported [14]. As for the respiratory component of the SOFA score, it is an integral assessment for acute respiratory distress syndrome (ARDS), which represents the development of diffuse and severe inflammation of pulmonary lobules in a short time causing hypoxemic respiratory failure [15]. However, the pathophysiologic association between ARDS and LDLT is unclear [16]. During the past decade, new therapeutic strategies for ARDS successfully decreased mortality [17]. The use of neuromuscular blocking agents in the acute phase of ARDS, the maturity of extracorporeal membrane oxygenation in ARDS support [17], and de-escalation of antibiotics in infectious lung conditions may all play a role in decreasing ARDS-associated mortality [18].

Among all SOFA components, the liver component showed the greatest discrimination, followed by the cardiovascular component (Table 3). The present study concluded that hyperbilirubinemia at a certain level (>5.9 mg/dL) at POD 7 indicated short-term mortality in LDLT recipients. In addition to the prolonged international normalized ratio on POD 7 and elevated alanine or aspartate aminotransferases within the first 7 days, poor resolution of serum hyperbilirubinemia is an essential diagnosis of early allograft dysfunction, the significance of which implies a devastating complication with substantial mortality [19]. The allograft dysfunction explains the rise in mortality in this group of patients. In the present model, the cut-off of cardiovascular component was set as a mean arterial pressure of less than 70 mmHg rather than inotropic use as previously described [20]. We believe that it reflects an early circulatory failure and subsequent cardiac failure. Shock is a status of acute circulatory failure with inadequate cellular oxygenation. Previous studies [21,22] reported that serum lactate level is a sensitive marker for shock status detection. From our data, multi-organ derangement in most graft failure cases were owing to infectious complications. Uncontrolled infection in the specific context of patients is probably an expression of the severity of their immune breakdown. Post-transplant infectious complications are estimated to occur in over half of recipients, especially in the early period, and bacterial pathogens account for the majority, followed by viral and fungal-origins [23,24].

Compared to other outcome prediction models [6,25,26], our GRWR-SOFA model was simplified and designed for risk prediction of short-term mortality after LDLT, exclusively. Therapeutic effects can be assessed through SOFA score over time, therefore allowing timely medical adjustment and helping to improve transplant results [27,28]. However, trends in serial SOFA assessment and dynamic application still require thorough investigation in post-transplant care.

Our model is very much a risk stratification tool that helps clinicians more systematically evaluate patients and assess the exact risk in developing early mortality. From a practical point, the implementation of the model is mainly in guiding timely medical adjustment in addition to the posttransplant care bundle regarding infection prevention, immunosuppressant dosing, hemodynamic stabilization, and strategy to prevent or reverse existed organ failure. For patients at high risk, with an associated 3-month mortality rate of 61.3%, an extension of ICU care, prolonged ventilator dependence, early renal replacement intervention, extra caution in bleeding tendency, adequate blood products transfusion, avoiding overt immunosuppression, application of broad-spectrum prophylactic antibiotics against potential bacterial and fungal infection, and early specialty consultation for a more comprehensive evaluation might improve the odds of surviving.

This study has the following limitations. First, it was retrospective in nature. Second, we set only a one-time point (POD 7) for SOFA assessment rather than dynamic evaluations. Third, our results were derived from a single-center experience. Concerning future developments, our results must be carefully interpreted before proving validated by future high-volume, prospective, and serial studies based on different populations.

In conclusion, the GRWR-SOFA model demonstrates superior discriminatory power for predicting short-term mortality. It emphasizes the importance of GRWR and validates the efficacy of SOFA scores on early post-transplant outcomes. This model is a simple and objective stratified scoring system that enables clinicians to identify the right level of care for distinct subgroups of patients undergoing LDLT.

## Figures and Tables

**Table 1:** Baseline demographics of 519 patients underwent LDLT.

General Information	Mean ± SD or Median (Minimun–Maximum Values)
Recipient age, year-old	53.9 ± 8.8 (19.1–70.2)
Recipient BMI, kg-m^−2^	25.0 ± 3.8 (16.4–42.1)
Recipient gender, male	388, 74.8%
Child–Pugh classification (B/C)	198/198, 38.2/38.2%
MELD score	16.9 ± 8.9 (8–40)
Viral hepatitis either B or C (yes)	382, 73.6%
Alcoholic (yes)	119, 22.9%
HCC (yes)	227, 43.7%
Transplant parameters	
Donor age, year-old	32.3 ± 9.2 (18.1–59.3)
Donor BMI, kg/m^2^	22.7 ± 2.8 (16.0–32.7)
Donor gender, male	226, 50.4%
Graft lobe (Right)	481, 92.7%
GRWR, %	0.98 ± 0.25 (0.51–2.02)
Ascites, mL	425 (0–28,800)
Intraoperative blood loss, mL	1625 (50–15,500)
Cold ischemic time, minutes	28 (5–246)
Warm ischemic time, minutes	36(15–232)
OP time, minutes	630.9 ± 140.0 (428–1219)
SOFA categories at POD 7	
Total SOFA score	5 (0–16)
Cardiovascular	0 (0–3)
Coagulation	2 (0–4)
Respiratory	1 (0–4)
Renal	0 (0–4)
Liver	2 (0–4)
Neurologic	0 (0–4)

Abbreviation: LDLT, living donor liver transplant; MELD, Model for End-Stage Liver Disease; GRWR, Graft recipient weight ratio; POD, postoperative day; OP, operation; BMI, body mass index; HBV, hepatitis B virus; HCV, hepatitis C virus; HCC, hepatocellular carcinoma; SOFA, Sequential Organ Failure Assessment.

**Table 2:** A comparative study (demographics) according to 3-month survival after transplant.

General Information	Survival, *n* = 477	Non-Survival, *n* = 42	*p* Value
Recipient age, year-old	53.9 ± 8.7	54.0 ± 10.8	0.953
Recipient BMI, kg-m^−2^	25.1 ± 3.8	24.6 ± 3.6	0.418
Recipient gender, male	360, 75.5%	28, 66.7%	0.208
Child–Pugh classification (B/C)	176/180, 36.9/37.7%	22/18, 52.4/42.9%	**0.008**
MELD score	16.5 ± 8.7	20.8 ± 10.6	**0.013**
Viral hepatitis infection (yes)	358, 75.1%	24, 57.1%	**0.012**
Alcoholic (yes)	106, 22.2%	13, 31.0%	0.197
HCC (yes)	217, 45.5%	10, 23.8%	**0.007**
Transplant parameters			
Donor age, year-old	32.0 ± 9.0	36.6 ± 9.8	**0.002**
Donor BMI, kg/m^2^	22.7 ± 2.8	23.4 ± 2.9	0.152
Donor gender, male	13.5 ± 12.1	11.7 ± 13.6	0.515
ABO compatibility, incompatible	80, 16.8%	5, 11.9%	0.414
GRWR, %	0.99 ± 0.25	0.87 ± 0.19	**0.001**
<0.8	107, 22.4%	20, 47.6%	**<0.001**
Ascites, mL	350 (0–28,800)	1800 (0–18,000)	**0.056**
>3000	121, 25.4%	20, 47.6%	0.002
Intraoperative blood loss, mL	1550 (50–15,500)	2400 (200–14,500)	**0.026**
>3000	92, 19.3%	15, 35.7%	**0.012**
Cold ischemic time, minutes	27 (5–246)	38 (8–228)	**0.260**
>120	15, 3.1%	4, 9.5%	0.035
Warm ischemic time, minutes	36 (15–232)	34 (24–64)	0.248
>60	13, 2.7%	3, 7.1%	0.112
OP time, minutes	628.4 ± 100.2	659.6 ± 137.9	0.159
>720	71, 14.9%	10, 23.8%	0.127
SOFA score and corresponding components scores at POD 7			
Total SOFA score	5 (0–15)	9 (2–16)	**<0.001**
>7	72, 15.1%	30, 71.4%	**<0.001**
Cardiovascular >0	17, 3.6%	7, 16.7%	**<0.001**
Coagulation >2	203, 42.6%	32, 76.2%	**<0.001**
Respiratory >1	123, 25.8%	15, 35.7%	0.163
Renal >0	99, 20.8%	26, 61.9%	**<0.001**
Liver >2	93, 19.5%	35, 83.3%	**<0.001**
Neurologic >0	40, 8.4%	14, 33.3%	**<0.001**

Abbreviation: MELD, Model for End-Stage Liver Disease; GRWR, Graft recipient weight ratio; OP, operation; POD, postoperative day; BMI, body mass index; HBV, hepatitis B virus; HCV, hepatitis C virus; HCC, hepatocellular carcinoma; SOFA, Sequential Organ Failure Assessment.

**Table 3:** Univariate and multivariate analyses to predict mortality within 3 months after transplant by logistic regression model.

		UV				MV	
	HR	95%CI	*p*-Value	HR	95% CI	β	*p*-Value
Non-HCC	2.67	1.28–5.56	0.009				
Non-Viral hepatitis	2.26	1.18–4.30	0.013				
MELD score > 20	1.73	0.90–3.34	0.099				
GRWR < 0.8	3.14	1.65–5.98	<0.001	3.11	1.40–6.89	1.134	**0.005**
Ascites > 3000 mL	2.68	1.41–5.07	0.003				
Blood loss > 3000 mL	2.33	1.19–4.55	0.014				
Cold ischemic time > 120 min	3.24	1.03–10.25	0.045				
SOFA scores at POD 7							
Cardiovascular > 0	5.41	2.10–13.92	<0.001	5.31	1.62–17.49	1.670	**0.006**
Coagulation > 2	4.32	2.08–8.99	<0.001	2.64	1.12–6.21	0.970	**0.026**
Renal > 0	6.21	3.20–12.02	<0.001	2.69	1.23–5.86	0.989	**0.013**
Liver > 2	20.65	8.89–47.94	<0.001	9.63	3.92–23.66	2.265	**<0.001**
Neurologic > 0	5.46	2.66–11.21	<0.001	2.75	1.15–6.61	1.012	**0.024**

Abbreviation: UV, univariate; MV, multivariate; HR, hazard ratio; CI, confidence interval; HCC, hepatocellular carcinoma; SOFA, Sequential Organ Failure Assessment Score; POD, postoperative day; GRWR, graft to recipient weight ratio. The regression coefficients (β) were multiplied by two and rounded to an integer in order to calculate further novel score.

**Table 4:** Constitution and risk classification of GRWR-SOFA Model.

Constitution of GRWR-SOFA Model		
Variables	Condition	Score allocation
Cardiovascular	MAP < 70mmHg	3
Coagulation	PLT < 50 × 10^3^/μL	2
Renal	Cr > 1.2 mg/dL	2
Liver	TB > 5.9 mg/dL	5
Neurologic	GCS < 15	2
GRWR	<0.8%	2
GRWR-SOFA Class obtained by adding score for each variable		
Class	Risk	Sum of six variables
I	Very low	0–4
II	Low	5–8
III	Intermediate	9–10
IV	High	≥11

Abbreviation: GRWR, graft-to-recipient weight ratio; SOFA, sofa sequential organ failure assessment; MAP, mean arterial pressure; PLT, platelet; Cr, creatinine; TB, total bilirubin; GCS, Glasgow coma scale. The prediction model was established by independent risk factors identified in multivariate analysis. The regression coefficients (β) were multiplied by two and rounded to an integer in order to calculate given score allocation.

## Data Availability

The data presented in this study are available on request from the corresponding author.

## Supplementary Materials

The following are available online at https://www.mdpi.com/article/10.3390/jcm10092014/s1, Figure S1: The performance of sequential organ failure assessment (SOFA) score at postoperative day 7 to predict 3-month mortality., Figure S2: Kaplan–Meier plot of 3-month overall survival according to GRWR-SOFA scores in sequence., Table S1: The distribution of SOFA score at POD 7 in various vital systems., Table S2: Patients distribution and survival according to GRWR-SOFA score and classification., Table S3: Distribution of GRWR-SOFA model reclassifies subjects—as compared to SOFA model.
