# Supplementary material for: Developing a Novel Scoring System for Risk Stratification in Living Donor Liver Transplantation
Source: J Clin Med. 2021 May 8;10(9):2014. doi: 10.3390/jcm10092014 (PMC8125826; doi:10.3390/jcm10092014)
Supplement: Supplementary file 1 [file jcm-10-02014-s001.zip › jcm-1166075-SI.pdf]

Supplementary File

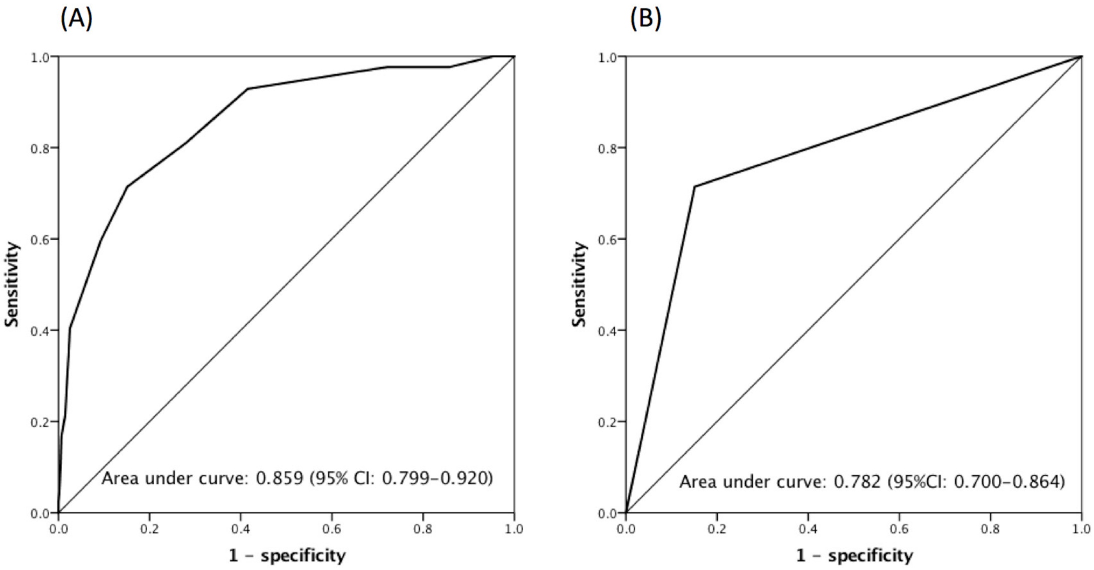

Figure S1. (A)The performance of sequential organ failure assessment (SOFA) score at postoperative day 7 to predict 3-month mortality is illustrated with an area under curve (AUC) of 0.859 (95% confidence interval [CI]: 0.799–0.920). (B) When an optimal cut-off value of 7 is used, it demonstrates an AUC of 0.782 (95% CI: 0.700–0.854).

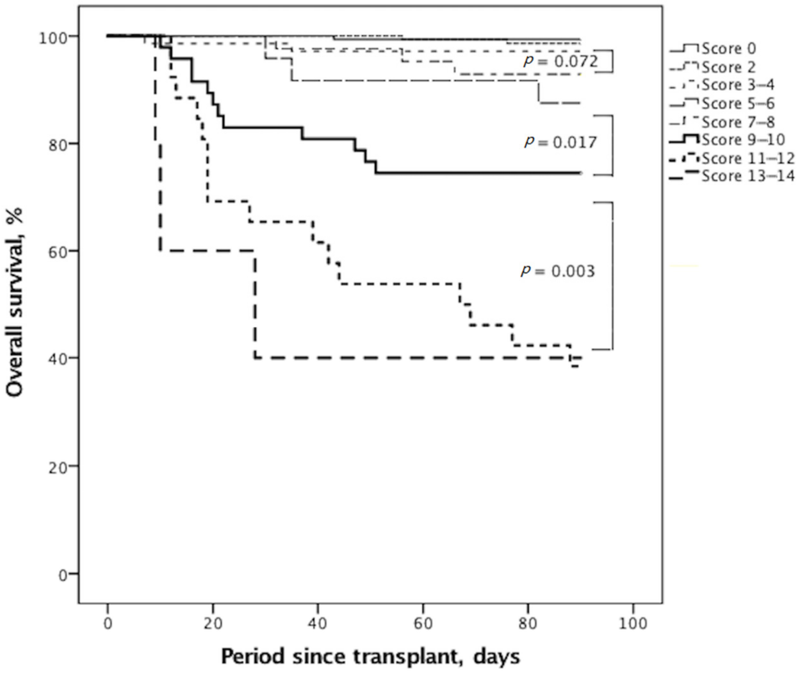

Figure S2. Kaplan–Meier plot of 3-month overall survival according to eight groups by every two graft-to-recipient weight ratio-sequential organ failure assessment (GRWR-SOFA) score in sequence (0, 2, 3–4, 5–6, 7–8, 9–10, 11–12, and 13–14). There is no survival difference ($p < 0.1$) between each group and its neighbor group except for the three matches (score 3–4 vs. 5–6, score 7–8 vs. 9–10, and score 9–10 vs. score 11–12), which is the foundation for further GRWR-SOFA classification.

Table S1. The distribution of SOFA score at POD 7 in various vital systems.

SOFA Components	Score, No. (%) 0	1	2	3	4
Cardiovascular	495 (95.4%)	21 (4.0%)	1 (0.2%)	2 (0.4%)	0 (0.0%)
Coagulation	21 (4.0%)	53 (10.2%)	210 (40.5%)	212 (40.8%)	23 (4.4%)
Renal	394 (75.9%)	69 (13.3%)	36 (6.9%)	10 (1.9%)	10 (1.9%)
Liver	142 (27.4%)	100 (19.3%)	149 (28.7%)	79 (15.2%)	49 (9.4%)
Neurologic	465 (89.6%)	24 (4.6%)	17 (3.3%)	10 (1.9%)	3 (0.6%)
Respiratory	220 (42.4%)	161 (31.0%)	113 (21.8%)	19 (3.7%)	6 (1.2%)

Abbreviation: SOFA, sequential organ failure assessment; POD, postoperative day;

Table S2. Patients distribution and survival according to GRWR-SOFA score and classification.

Patients' distribution and survival		
Total score	Case No. (%)	3-month mortality (%)
0	160 (30.8%)	1 (0.63%)
2	145 (27.9%)	2 (1.38%)
3–4	70 (13.5%)	2 (2.86%)
5–6	24 (4.6%)	3 (12.5%)
7–8	42 (8.1%)	3 (7.14%)
9–10	47 (9.1%)	12 (25.0%)
11–12	26 (5.0%)	16 (61.5%)
13–14	5 (1.0%)	3 (60.0%)
15–16	0 (0.0%)	NA
The GRWR-SOFA classification		
Class (adding score)	Case No. (%)	3-month mortality (%)
Class I (0–4)	375 (72.3%)	5 (1.3%)
Class II (5–8)	66 (12.7%)	6 (9.1%)
Class III (9–10)	47 (9.1%)	12 (25.5%)
Class IV (≥11)	31 (6.0%)	19 (61.3%)

Abbreviation: GRWR, graft to recipient weight ratio; SOFA, sofa sequential organ failure assessment; No, number; NA, not applicable.

Table S3. Distribution of GRWR-SOFA model reclassifies subjects - as compared to SOFA model.

Non-survivor, *n* / Survivor, *n*		GRWR-SOFA classification I	II	III	IV	Total, split	Total
SOFA	≤7	4	3	4	1	12	417
		350	36	17	2	405	
	>7	1	3	8	18	30	102
		20	24	18	10	72	
Total, split		5	6	12	19	42	
		370	60	35	12	477	
Total		375	66	47	31		519

Abbreviation: SOFA, sequential organ failure assessment; GRWR, graft-recipient weight ratio.
